# Impact of reduced exposure to calcineurin inhibitors on the development of de novo DSA: a cohort of non-immunized first kidney graft recipients between 2007 and 2014

**DOI:** 10.1186/s12882-018-1014-2

**Published:** 2018-09-15

**Authors:** S. Girerd, J. Schikowski, N. Girerd, K. Duarte, H. Busby, N. Gambier, M. Ladrière, M. Kessler, L. Frimat, A. Aarnink

**Affiliations:** 1 Service de Néphrologie et Transplantation rénale, CHRU Nancy Brabois, Vandoeuvre-les-, Nancy, France; 2 INSERM, Centre d’Investigations Cliniques Plurithématique 1433, Université de Lorraine, CHRU de Nancy and F-CRIN INI-CRCT, Nancy, France; 3 Service d’Anatomie pathologique, CHRU Nancy Brabois, Vandœuvre-lès-Nancy, France; 4 Service de Pharmacologie-Toxicologie, CHRU Nancy Brabois, Vandœuvre-lès-Nancy, France; 5 Laboratoire d’Histocompatibilité, CHRU Nancy Brabois, Vandœuvre-lès-Nancy, France

**Keywords:** Kidney transplantation, Calcineurin inhibitors, Donor specific antibodies, Under immunosuppression

## Abstract

**Background:**

In low-immunological risk kidney transplant recipients (KTRs), reduced exposure to calcineurin inhibitor (CNI) appears particularly attractive for avoiding adverse events, but may increase the risk of developing de novo Donor Specific Antibodies (dnDSA).

**Methods:**

CNI exposure was retrospectively analyzed in 247 non-HLA immunized first KTRs by taking into account trough levels (C0) collected during follow-up. Reduced exposure to CNI was defined as follows: C0 less than the lower limit of the international targets for ≥50% of follow-up.

**Results:**

During a mean follow-up of 5.0 ± 2.0 years, 39 patients (15.8%) developed dnDSA (MFI ≥1000). Patients with DSA were significantly younger (46.6 ± 13.8 vs. 51.7 ± 14.0 years, *p* = 0.039), received more frequently poorly-matched grafts (59% with 6–8 A-B-DR-DQ HLA mismatches vs. 34.6%, *p* = 0.016) and had more frequently a reduced exposure to CNI (92.3% vs. 62.0%, *p* = 0.0002). Reduced exposure to CNI was associated with an increased risk of dnDSA (multivariable HR = 9.77, *p* = 0.002)*.* Reduced exposure to CNI had no effect on patient survival, graft loss from any cause including death, or post-transplant cancer.

**Conclusions:**

Even in a low-immunological risk population, reduced exposure to CNI is associated with increased risk of dnDSA. Benefits and risks of under-immunosuppression must be carefully evaluated before deciding on CNI minimization.

## Background

Calcineurin inhibitors (CNI) were first introduced in the 1980s and have led to dramatic improvements in short-term kidney transplantation outcomes. Nevertheless, CNI were traditionally thought to be the major contributors of chronic kidney graft dysfunction due to nephrotoxicity [1]. This historical view was challenged during the past decades [2, 3], given that chronic graft nephropathy was largely related to humoral chronic rejection [4–6] and not only CNI nephrotoxicity [7].

Nevertheless, the overall level of immunosuppressive therapy obviously increases the risk of infectious or neoplastic complications [8]. Therefore, clinicians continue to attempt numerous protocols to reduce exposure to CNI, including primary avoidance, dose reduction, and switching to other drug classes, namely mTOR inhibitors or belatacept [9–12].

There is now a large body of evidence whereby antibody-mediated rejection (ABMR) is the major cause of late kidney allograft failure [4–6]. CNI minimization may fail to improve long-term outcomes due to the development of Donor Specific Antibodies (DSA) and chronic rejection despite less chronic nephrotoxicity. Thus, nephrotoxicity prevention by CNI minimization may be counterbalanced by an increased risk of DSA development, leading to non-significant improvements in long-term graft prognosis. In low immunological risk populations, the impact of reduced exposure to CNI is of particular interest, considering that the benefit/risk balance is, a priori, in disfavor of strong immunosuppressive therapy.

The present study aimed to assess the impact of reduced exposure to CNI (i.e. CNI trough level reduction without avoidance or switch) on the development of de novo DSA (dnDSA) among a cohort of low-immunological risk patients, i.e. first kidney transplant recipients (KTRs) with negative class I and class II anti-HLA antibodies prior to transplantation.

## Methods

### Study population

**Fig. 1:**
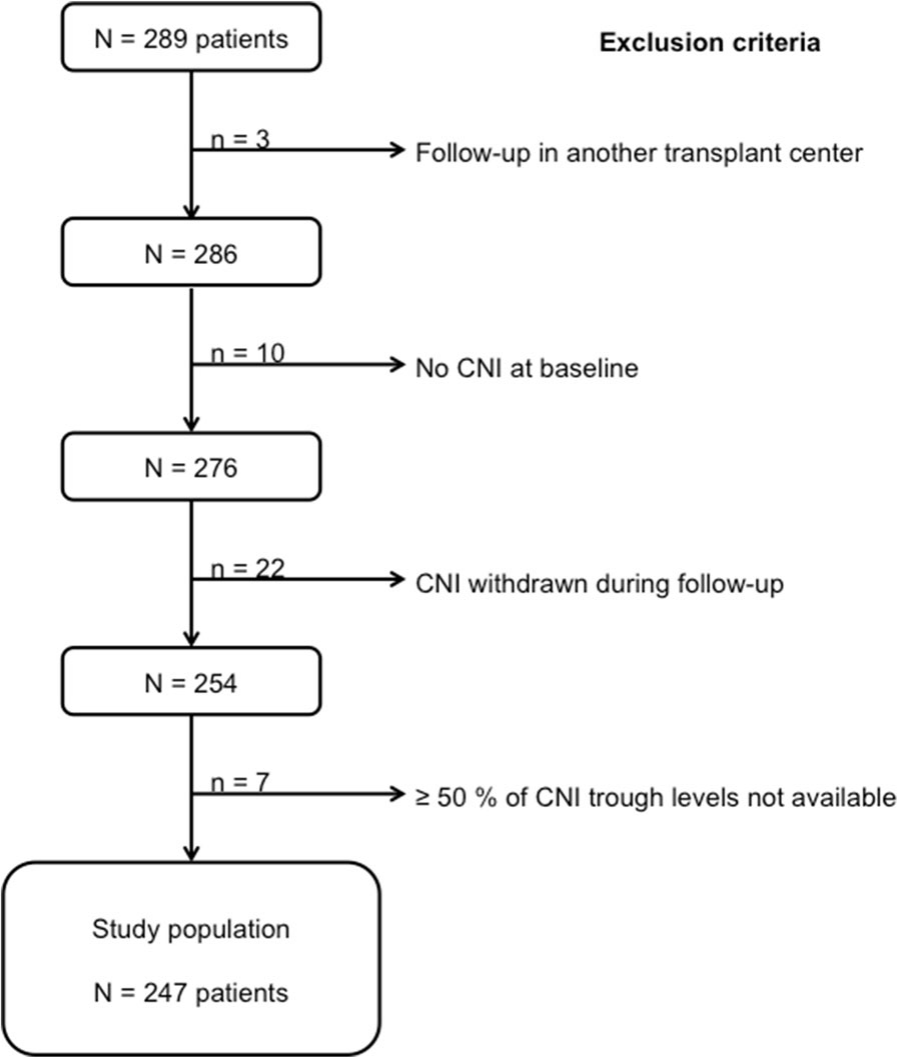
Flow chart of the study population

This observational single-center cohort study included all non-immunized first KTRs in the University Hospital of Nancy between 01/01/2007 and 31/12/2014. Exclusion criteria consisted of patients aged < 18 years, receiving a combined non-renal graft, or followed in another center after the transplantation. Patients who did not receive CNI or had CNI discontinuation during follow-up were also excluded. Patients with more than 50% of missing values of CNI trough levels (*n* = 7) were also excluded. The study population flowchart is presented in Fig. 1. Non-immunization was defined by the absence of both class I and class II anti-HLA antibodies before transplantation as assessed by Luminex technique, as described hereafter.

Immunosuppressive therapy consisted in an induction therapy (anti-thymocyte globulins or anti-IL2 monoclonal antibody), steroid pulses, followed by maintenance therapy generally including long-term oral corticotherapy (5 mg/day), an antimetabolite (mycophenolic acid or azathioprine) and CNI (either tacrolimus or cyclosporine). The usual initial dosage of tacrolimus was 0.15 mg/kg/day for tacrolimus and 6 mg/kg/day for cyclosporine. The initial dosage of mycophenolic acid was 1000 mg/day when associated with tacrolimus and 2000 mg/day when associated with cyclosporine.

### Data collection

Data were extracted from the prospective French database of transplanted patients DIVAT (computerized and VAlidated data in Transplantation) (www.divat.fr). Written informed consent was obtained from all participants and The “Comité National de l’Informatique et des Libertés” approved the study (CNIL no. 891735). Data were entered in a computerized database on day 0, at 3 months and 12 months, and subsequently updated annually thereafter. Patients were followed annually until June 2016.

Characteristics collected at baseline included: age, gender, body mass index, comorbidities, causal nephropathy, dialysis method and time on dialysis prior to kidney transplantation, as well as duration on waiting list. Transplantation parameters included: donor type (living donors; standard criteria donors (SCD); expanded criteria donors (ECD) defined as follows: donors aged ≥60 years, or donors aged 50–59 years with ≥2 of the following conditions: history of hypertension, cerebrovascular cause of death, serum creatinine greater than 1.5 mg/dL), cold ischemia time, HLA A-B-DR-DQ incompatibilities, induction therapy and maintenance immunosuppressive regimen, as well as delayed graft function defined by the necessity of one or more dialysis sessions in the first week after transplantation. Data collected during follow-up included: dnDSA detection, acute rejection, return to dialysis and death before return to dialysis. Post-transplant cancers were also recorded. Patients were followed until death, return to dialysis or last follow-up visit up until March 2016. Mean follow-up was 5.0 ± 2.0 years.

#### Anti-HLA immunization and DSA detection

All patients included in the study cohort underwent a search for anti-HLA class I and class II antibodies that was negative prior to transplantation. The monitoring of anti-HLA immunization after transplantation was performed at 3, 6 and 12 months after the graft and annually thereafter, as well as at the time of biopsy when clinically warranted (presence of graft dysfunction or suspicion of rejection). The sera were screened for HLA-specific antibodies using solid-phase Luminex HLA antibody-detection beads (LABScreen™ Mixed, One Lambda Inc., Canoga Park) and selected HLA-specific antibody-positive samples were analyzed using Luminex single-antigen HLA class I and class II antibody-detection beads (LABScreen™ Single Antigen HLA Class I and Class II, One Lambda Inc., Canoga Park). Antibodies were considered as Donor Specific Antibodies if the MFI (Mean Fluorescence Intensity) of antibodies directed against a donor antigen (HLA-A, -B, -C, −DR, −DR51, −DR52, −DR53, −DQ or -DP) was greater than 1000. For each serum, the sum of MFI DSA(s) was also studied. In instances where the recipient had DSA directed against a homozygous donor antigen, the MFI was doubled.

#### Exposure to CNI

**Fig. 2:**
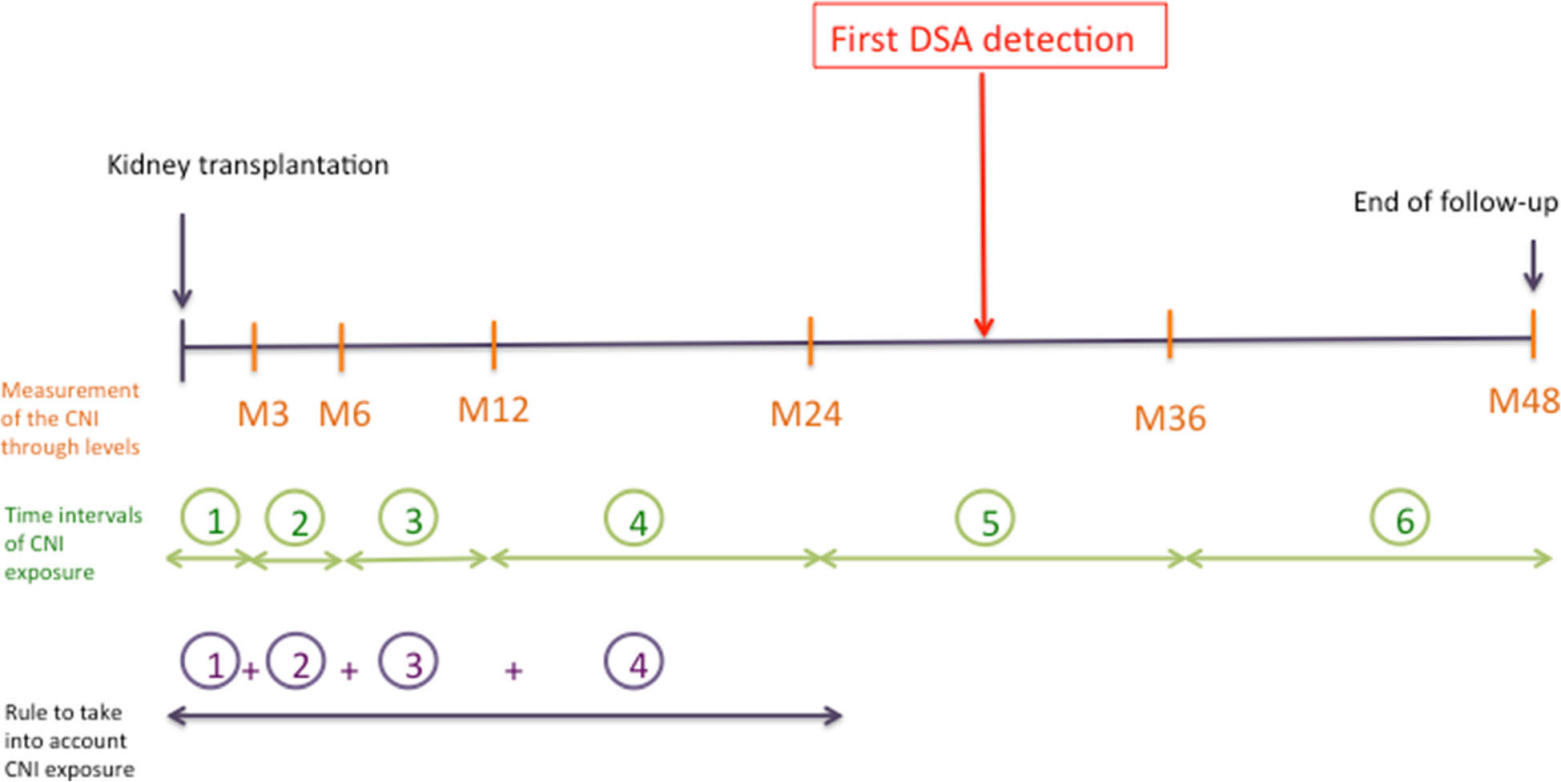
Method used to take into account CNI exposure prior to the event of interest (DSA onset, rejection, return to dialysis, death)

The blood concentration of CNI were measured by the antibody-conjugated magnetic immunoassay (ACMIA) method using an Dimension® system (Siemens). The lower limit of quantification was 25 ng/mL and 2 ng/mL for cyclosporine and tacrolimus, respectively. Trough levels were measured at month 3, month 6, month 12, and annually thereafter. For every patient and every outcome, the number of time intervals of CNI exposure before the event was established considering that the event itself could lead to a modification in CNI posology (the next trough level may be the consequence of the event). For example, in the case of first DSA detection at month 30 (Fig. 2), the time intervals M0-M3 (trough level measured at month 3), M3-M6 (trough level measured at month 6), M6-M12 (trough level measured at month 12) and M12-M24 (trough level measured at month 24) were taken into account.

**Table:** 

Post transplant delay	Cyclosporine trough level (ng/mL)	Tacrolimus trough level (ng/mL)
0–3 months	250–350	10–15
3–6 months	150–250	8–10
6–12 months	125–200	6–8
> 12 months	100–150	5–8

Trough levels less than the lower limit of the international targets [13–15] for ≥50% of time intervals defined the reduced exposure to CNI. In case of a missing value, the time interval was not considered, and the total number of intervals decreased accordingly.

Consequently, patients were classified into two groups according to the presence or the absence of a reduced exposure to CNI. Patients having developed dnDSA or not during follow-up were also compared.

**Fig. 3:**
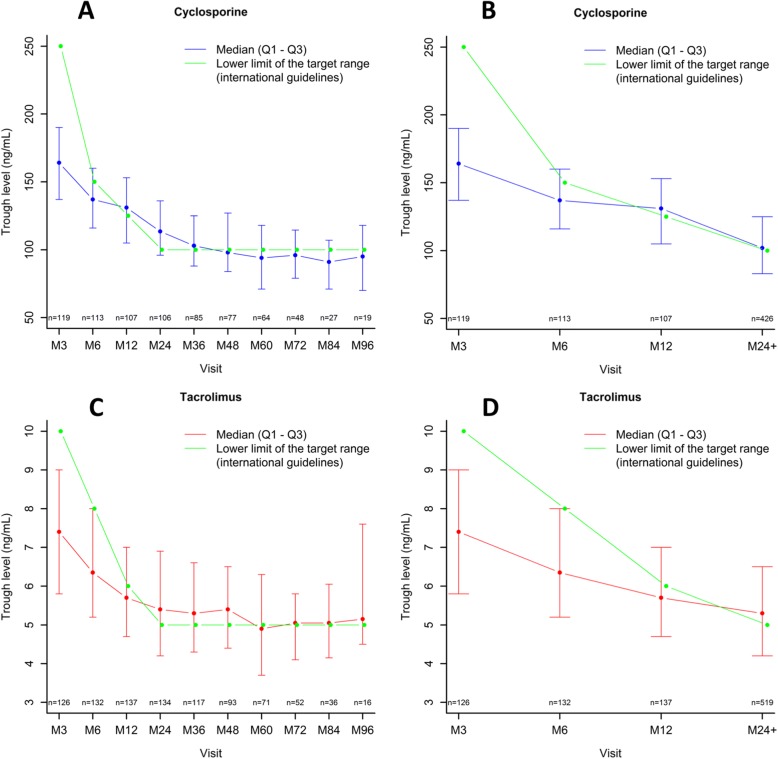
Distribution of CNI trough levels of the study population along with the lower limit of the target range according to international guidelines. Dots represent the median value of CNI through levels and vertical bars depict interquartile ranges. **a** for cyclosporine during the entire follow-up. **b** for cyclosporine during the first two years of transplantation. **c** for tacrolimus during the entire follow-up. **d** for tacrolimus during the first two years of transplantation

The distribution of CNI trough levels at each visit is presented in Fig. 3 a-d. The number of patients recieving tacrolimus increased over time while the number of patients receiving cyclosporin decreased, because some patients were switched from cyclosporin to tacrolimus during follow-up (Fig. 3).

### Statistical methods

All analyses were performed using R software (the R foundation for Statistical Computing). The two-tailed significance level was set at *p* < 0.05. Continuous variables are described as means ± standard deviation, categorical variables as frequencies (percentages). Hazard ratios are presented with their 95% confidence intervals as HR (CI 95%). Comparisons of baseline characteristics according to reduced exposure to CNI or not or DSA detection were carried out using the non parametric Mann-Whitney test for continuous variables and chi-square or Fisher’s exact test for categorical variables. Time-to-event analyses using Cox regression models were performed to assess the associations between reduced exposure to CNI and outcomes (DSA detection, return to dialysis, death before return to dialysis, return to dialysis or death before return to dialysis). Proportional hazard assumption was thoroughly verified using the Schoenfeld residuals test. Multivariable analyses were performed using iterative backward selection (*p* < 0.05), by forcing “reduced exposure to CNI” in the Cox model, with the following variables as candidate covariates: number of HLA mismatches, donor type, age and gender of the recipient, mycofenolic acid cessation, delayed graft function and induction therapy. Survival rates are illustrated using Kaplan Meier analyses. Differences between survival curves were analyzed using the log-rank test. Intra-patient variability (IPV) of CNI trough levels was calculated. As others [16], C0 blood levels of cyclosporine to C0 tacrolimus equivalents were converted using an empiric 1/20 correction factor, based on the limits of the international targets.

Intra-patient variability (IPV) in CNI exposure was also studied and defined as the fluctuation in CNI blood concentrations within an individual over a given period. The mean absolute deviation percent (MAD%) formula was used as previously described [17]:

$$ MAD\%=\frac{1}{n}\sum \frac{abs\left( Xj-\overline{X}\right)}{\overline{X}} $$* 100.

where:

- $\overline{X}$ represents the average of all available samples (in the case of tacrolimus IPV, the average of all tacrolimus trough levels measured for time period j), X_j_ represents an individual data point (a single tacrolimus trough level measurement) and n the number of all available data points (the total number of all available tacrolimus trough levels during period j)

- Abs (…) denotes the absolute value function, such that the quantitative value $abs\left( Xj-\overline{X}\right)$is always a non-negative value. The obtained tacrolimus trough level (C0) must be corrected to the corresponding daily tacrolimus dose (C0/D)

## Results

### Baseline characteristics of the entire population

A total of 247 KTRs were included. Mean age of graft recipients was 50.9 ± 14.1 years. The proportion of living donors was 27.5% while 15.4% of patients received preemptive kidney transplantation. With regard to HLA compatibility, 24.7% patients had 0–3 mismatches (A-B-DR-DQ), 36.8% had 4–5 mismatches and 38.5% had 6–8 mismatches. An induction treatment was administered in 95.1% of patients (70.2% with lymphocyte-depletive agent and 29.8% with anti-interleukin-2 receptor antibodies), while 49.0% received cyclosporine as maintenance therapy with the remaining 51.0% receiving tacrolimus. Other baseline data are provided in Table 1*.*

**Table 1:** Baseline and follow-up data of patients according to the absence or presence of a reduced exposure to CNI

	Total (*n* = 247)	Controls (*n* = 82)	Reduced exposure to CNI (*n* = 165)	*p*-value
**DEMOGRAPHICS**				
Age (years)	50.9 ± 14.1	51.1 ± 13.1	50.8 ± 14.6	0.91
Male (n, %)	173 (70.0%)	62 (75.6%)	111 (67.3%)	0.18
BMI (kg/m^2^)	25.5 ± 4.8	26.2 ± 4.3	25.1 ± 4.9	0.026
**COMORBIDITIES**				
Hypertension (n, %)	235 (95.1%)	77 (93.9%)	158 (95.8%)	0.54
No smoker (n, %)	122 (49.4%)	44 (53.7%)	78 (47.3%)	
Former smoker (n, %)	93 (37.7%)	28 (34.1%)	65 (39.4%)	
Active smoker (n, %)	32 (13.0%)	10 (12.2%)	22 (13.3%)	
Stroke (n, %)	11 (4.5%)	5 (6.1%)	6 (3.6%)	0.51
Diabetes (n, %)	55 (22.3%)	20 (24.4%)	35 (21.2%)	0.57
Type 1 diabetes	13 (23.6%)	3 (15.0%)	10 (28.6%)	
Type 2 diabetes (with insulin)	40 (72.7%)	17 (85.0%)	23 (65.7%)	
Type 2 diabetes (no insulin)	2 (3.6%)	0 (0.0%)	2 (5.7%)	
History of coronary disease (n, %)	25 (10.1%)	9 (11.0%)	16 (9.7%)	0.75
Heart failure (n, %)	41 (16.6%)	17 (20.7%)	24 (14.5%)	0.22
Peripheral artery disease (n, %)	19 (7.7%)	4 (4.9%)	15 (9.1%)	0.24
Chronic obstructive pulmonary disease (n, %)	8 (3.2%)	5 (6.1%)	3 (1.8%)	0.12
Pre-transplant cancer (n, %)	10 (4.0%)	4 (4.9%)	6 (3.6%)	0.73
Causal nephropathy (n, %)				
Other	25 (10.1%)	4 (4.9%)	21 (12.7%)	0.27
Chronic glomerulonephritis	63 (25.5%)	21 (25.6%)	42 (25.5%)	
Toxic	2 (0.8%)	1 (1.2%)	1 (0.6%)	
Diabetic nephropathy	37 (15.0%)	10 (12.2%)	27 (16.4%)	
Vascular nephropathy	20 (8.1%)	9 (11.0%)	11 (6.7%)	
Unknown	37 (15.0%)	14 (17.1%)	23 (13.9%)	
Polycystic disease	45 (18.2%)	13 (15.9%)	32 (19.4%)	
Nephrectomy	2 (0.8%)	1 (1.2%)	1 (0.6%)	
Malformative uropathy	12 (4.9%)	6 (7.3%)	6 (3.6%)	
Vasculitis	4 (1.6%)	3 (3.7%)	1 (0.6%)	
Dialysis prior to transplantation (n, %)	209 (84.6%)	73 (89.0%)	136 (82.4%)	0.18
Peritoneal dialysis	38 (18.2%)	15 (20.5%)	23 (16.9%)	
Hemodialysis	171 (81.8%)	58 (79.5%)	113 (83.1%)	
Pre-transplant dialysis time (years)	2.1 ± 2.0	2.1 ± 2.0	2.2 ± 2.0	0.73
**TRANSPLANTATION DATA**				
Viral status CMV donor/recipient (n, %)				0.51
D−/R-	59 (23.9%)	22 (26.8%)	37 (22.4%)	
D−/R+	57 (23.1%)	18 (22.0%)	39 (23.6%)	
D+/R-	64 (25.9%)	17 (20.7%)	47 (28.5%)	
D+/R+	67 (27.1%)	25 (30.5%)	42 (25.5%)	
Donor type (n, %)				**0.0008**
Expanded criteria donor	63 (25.5%)	17 (20.7%)	46 (27.9%)	
Standard criteria donor	116 (47.0%)	52 (63.4%)	64 (38.8%)	
Living donor	68 (27.5%)	13 (15.9%)	55 (33.3%)	
Cold ischemia time (hours)	13.1 ± 8.9	14.5 ± 8.0	12.4 ± 9.3	**0.023**
HLA A-B-DR-DQ incompatibilities (n, %)				
0–3	61 (24.7%)	17 (20.7%)	44 (26.7%)	**0.048**
4–5	91 (36.8%)	39 (47.6%)	52 (31.5%)	
6–8	95 (38.5%)	26 (31.7%)	69 (41.8%)	
Induction treatment (n, %)	235 (95.1%)	77 (93.9%)	158 (95.8%)	0.54
Lymphocyte-depletive agent	165 (70.2%)	56 (72.7%)	109 (69.0%)	
Anti-interleukin-2 receptor antibodies	70 (29.8%)	21 (27.3%)	49 (31.0%)	
Mycofenolic acid cessation during follow-up	28 (11.3%)	9 (11.0%)	19 (11.5%)	0.90
**POST-TRANSPLANTATION EVENTS**				
Delayed graft function (n, %)	72 (29.1%)	30 (36.6%)	42 (25.5%)	0.070
Rejection (n, %)	42 (17.0%)	9 (11.0%)	33 (20.0%)	0.075
T cell mediated rejection	40 (16.2%)	8 (9.8%)	32 (19.4%)	
Antibody mediated rejection	3 (1.2%)	1 (1.2%)	2 (1.2%)	
Time to first rejection (years)	0.5 ± 1.0	0.9 ± 1.2	0.4 ± 0.9	
Post-transplant cancer (n, %)	29 (11.7%)	9 (11.0%)	20 (12.1%)	0.79
Skin cancer	11 (37.9%)	2 (22.2%)	9 (45.0%)	
Hemopathy	3 (10.3%)	1 (11.1%)	2 (10.0%)	
Solid cancer	15 (51.7%)	6 (66.7%)	9 (45.0%)	
Time to post-transplant cancer (years)	3.2 ± 1.9	4.0 ± 2.0	2.9 ± 1.8	0.15
Return in dialysis (n, %)	18 (7.3%)	3 (3.7%)	15 (9.1%)	0.12
Death with a functioning graft (n, %)	22 (8.9%)	9 (11.0%)	13 (7.9%)	0.42
Graft failure from any cause including death (n, %)	40 (16.2%)	12 (14.6%)	28 (17.0%)	0.64
CNI Mean Absolute Deviation (%)				
Continuous	19.9 ± 9.6	19.2 ± 10.3	20.3 ± 9.3	0.21
< 5%	6 (2.4%)	2 (2.4%)	4 (2.4%)	1.00
< 15%	87 (35.2%)	36 (43.9%)	51 (30.9%)	**0.044**
> 30%	32 (13.0%)	12 (14.6%)	20 (12.1%)	0.58
> 50%	2 (0.8%)	1 (1.2%)	1 (0.6%)	1.00

*BMI* Body Mass Index*, DSA* Donor Specific Antibody*, D−/R-* Donor negative/Recipient negative*, D−/R+* Donor negative/Recipient positive*, D+/R-* Donor positive/Recipient negative*, CNI* Calcineurin inhibitors. Results with *p* value less than 5% were emphasized using bold letters

### Baseline characteristics of patients according to “exposure to CNI” status

Patient characteristics according to the presence or the absence of a reduced exposure to CNI are presented in Table 1. Patients did not differ in terms of age, causal nephropathy or medical history (cancer or infectious disease prior to transplantation as well as cardiovascular history). Of note, the proportion of living donors and the proportion of expanded criteria donors were higher in the group with reduced exposure to CNI (respectively 33.3% vs. 15.9 and 27.9% vs. 20.7%, *p* = 0.0008). Moreover, the number of HLA A-B-DR-DQ incompatibilities differed according to the two groups with a higher proportion of very well matched patients as well as very poorly matched patients in the group with reduced exposure to CNI (26.7% vs. 20.7 and 41.8% vs. 31.7%, *p* = 0.048 for 0–3 mismatches and 6–8 mismatches, respectively). The proportion of induction treatment was similar in the two groups, as well as the proportion of mycofenolic acid cessation during follow-up. The proportion of patients with high CNI IPV was similar in the two groups (for IPV > 30%: 12.1% vs. 14.6%, *p* = 0.58).

### Follow-up data: DSA detection and impact of reduced exposure to CNI on DSA appearance

**Fig. 4:**
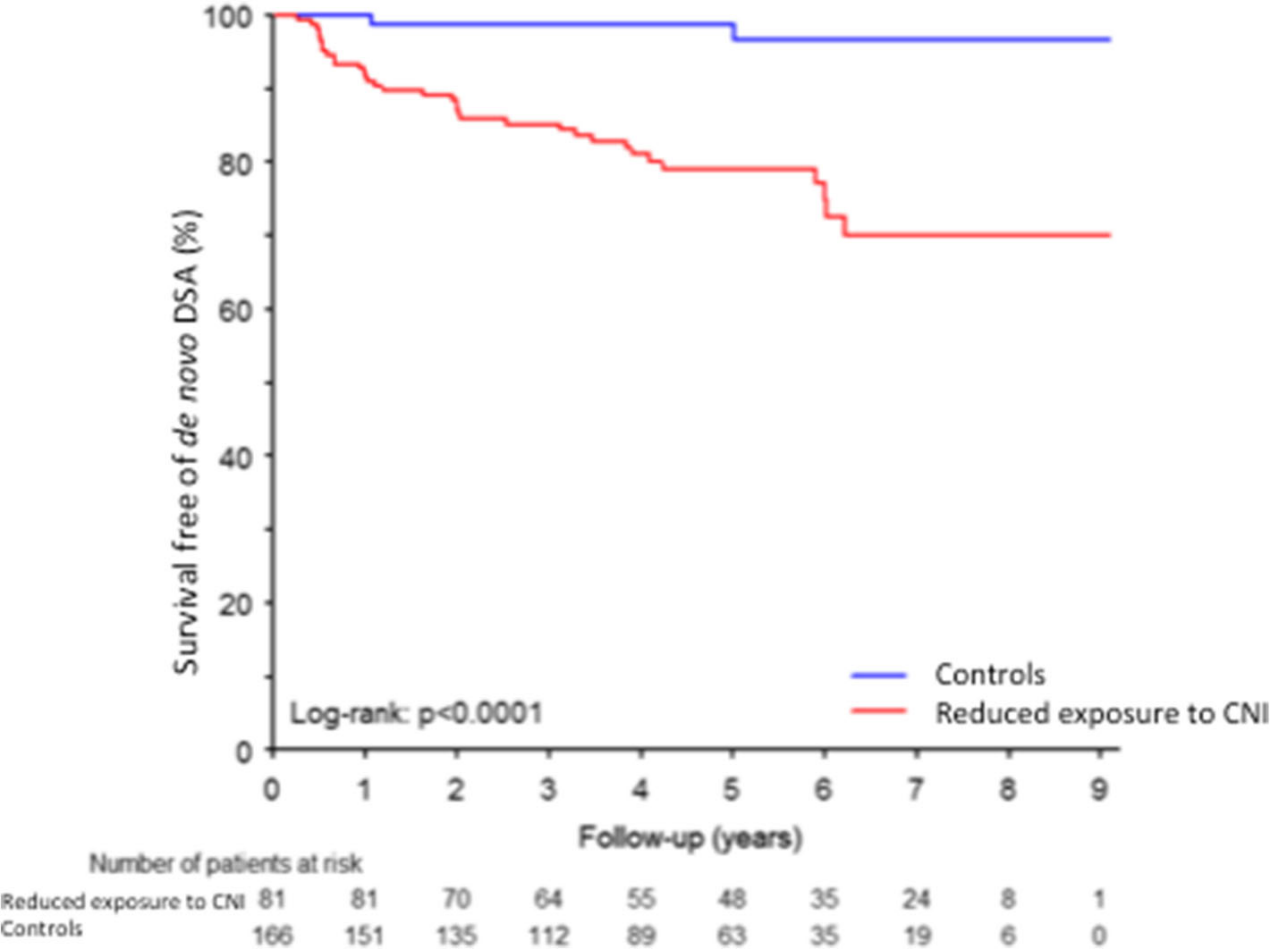
De novo DSA detection according to the presence or the absence of a reduced exposure to CNI

During follow-up, 39 patients (15.8%) developed dnDSA (with MFI ≥1000). The proportion of KTRs who developed DSA during follow-up was higher in the group of patients with reduced exposure to CNI (21.1% (35/166) vs 2.5% (2/81), *p* < 0.0001) (Fig. 4). Patients who developed dnDSA were significantly younger (46.6 ± 13.8 vs. 51.7 ± 14.0, *p* = 0.039) (Table 2), received more frequently poorly-matched grafts (59% with 6–8 HLA mismatches in the group with DSA vs. 34.6% in the group without DSA, *p* = 0.016), and have more frequently a reduced exposure to CNI (92.3% vs. 62.0%, *p* = 0.0002). The proportion of induction therapy was similar in both groups, as well as mycofenolic acid cessation during follow-up, or IPV. Of note, no patient had mycofenolic acid cessation before the first DSA detection in the group with DSA (Table 2). In a multivariate analysis adjusted for the number of HLA mismatches, donor type, age and gender of the recipient, mycofenolic acid cessation, delayed graft function and induction therapy, reduced exposure to CNI was associated with an increased risk of DSA development (for first detection of one DSA with MFI > 1000, HR in multivariable analysis 9.77 (2.34–40.77), *p* = 0.002; for first detection of DSAs with total MFI (sum MFI) > 6000 HR = 12.02 (1.62–89.25), *p* = 0.015) (Table 3). When adjusting, as a sensitivity analysis, on number HLA mismatches, donor type and induction treatment (without iterative backward selection) we found similar results (*data not shown*). When adjusting, as a sensitivity analysis, on number HLA mismatches, donor type and induction treatment (without iterative backward selection) we found similar results (*data not shown*). In addition, when adjusting on body mass index and cold ischemia time (without selection, based on the univariable analysis) we found similar associations and *p*-values (*data not shown*).

**Table 2:** Baseline and follow-up data of patients according to the absence or presence of de novo DSA during follow-up

	Population (*n* = 247)	No DSA (*n* = 208)	De novo DSA (*n* = 39)	*p*-value
**DEMOGRAPHICS**				
Age (years)	50.9 ± 14.1	51.7 ± 14.0	46.6 ± 13.8	**0.039**
Male (n, %)	173 (70.0%)	142 (68.3%)	31 (79.5%)	0.16
BMI (kg/m^2^)	25.5 ± 4.8	25.3 ± 4.9	26.4 ± 4.1	0.16
**COMORBIDITIES**				
No smoker (n, %)	122 (49.4%)	101 (48.6%)	21 (53.8%)	0.81
Former smoker (n, %)	93 (37.7%)	80 (38.5%)	13 (33.3%)	
Current smoker (n, %)	32 (13.0%)	27 (13.0%)	5 (12.8%)	
Stroke (n, %)	11 (4.5%)	11 (5.3%)	0 (0.0%)	0.22
Diabetes (n, %)	55 (22.3%)	44 (21.2%)	11 (28.2%)	0.33
Type 1 diabetes	13 (23.6%)	9 (20.5%)	4 (36.4%)	
Type 2 diabetes (with insulin)	40 (72.7%)	34 (77.3%)	6 (54.5%)	
Type 2 diabetes (no insulin)	2 (3.6%)	1 (2.3%)	1 (9.1%)	
History of coronary disease (n, %)	25 (10.1%)	21 (10.1%)	4 (10.3%)	1.00
Heart failure (n, %)	41 (16.6%)	33 (15.9%)	8 (20.5%)	0.47
Peripheral artery disease (n, %)	19 (7.7%)	16 (7.7%)	3 (7.7%)	1.00
Pre-transplant cancer (n, %)	10 (4.0%)	9 (4.3%)	1 (2.6%)	1.00
Causal nephropathy (n, %)				0.24
Other	25 (10.1%)	20 (9.6%)	5 (12.8%)	
Chronic glomerulonephritis	63 (25.5%)	52 (25.0%)	11 (28.2%)	
Toxic	2 (0.8%)	2 (1.0%)	0 (0.0%)	
Diabetic nephropathy	37 (15.0%)	27 (13.0%)	10 (25.6%)	
Vascular nephropathy	20 (8.1%)	19 (9.1%)	1 (2.6%)	
Unknown	37 (15.0%)	35 (16.8%)	2 (5.1%)	
Polycystic disease	45 (18.2%)	38 (18.3%)	7 (17.9%)	
Nephrectomy	2 (0.8%)	1 (0.5%)	1 (2.6%)	
Malformative uropathy	12 (4.9%)	10 (4.8%)	2 (5.1%)	
Vasculitis	4 (1.6%)	4 (1.9%)	0 (0.0%)	
Dialysis prior to transplantation (n, %)	209 (84.6%)	174 (83.7%)	35 (89.7%)	0.33
Peritoneal dialysis	38 (18.2%)	32 (18.4%)	6 (17.1%)	
Hemodialysis	171 (81.8%)	142 (81.6%)	29 (82.9%)	
Pre-transplant dialysis time (years)	2.1 ± 2.0	2.1 ± 1.9	2.2 ± 2.1	0.78
**TRANSPLANTATION DATA**				
Viral status CMV donor/recipient (n, %)				0.72
D−/R-	59 (23.9%)	47 (22.6%)	12 (30.8%)	
D−/R+	57 (23.1%)	48 (23.1%)	9 (23.1%)	
D+/R-	64 (25.9%)	55 (26.4%)	9 (23.1%)	
D+/R+	67 (27.1%)	58 (27.9%)	9 (23.1%)	
Donor type (n, %)				
Expanded criteria donor	63 (25.5%)	55 (26.4%)	8 (20.5%)	0.72
Standard criteria donor	116 (47.0%)	97 (46.6%)	19 (48.7%)	
Living donor	68 (27.5%)	56 (26.9%)	12 (30.8%)	
Cold ischemia time (hours)	13.1 ± 8.9	13.1 ± 8.5	12.8 ± 10.9	0.38
HLA A-B-DR-DQ incompatibilities (n, %)				**0.016**
0–3	61 (24.7%)	55 (26.4%)	6 (15.4%)	
4–5	91 (36.8%)	81 (38.9%)	10 (25.6%)	
6–8	95 (38.5%)	72 (34.6%)	23 (59.0%)	
Induction treatment (n, %)	235 (95.1%)	196 (94.2%)	39 (100.0%)	0.22
Lymphocyte-depletive agent	165 (70.2%)	136 (69.4%)	29 (74.4%)	
Anti-interleukin-2 receptor antibodies	70 (29.8%)	60 (30.6%)	10 (25.6%)	
Reduced exposure to CNI (n, %)	165 (66.8%)	129 (62.0%)	36 (92.3%)	**0.0002**
Mycophenolic acid cessation				
During the entire follow-up	28 (11.3%)	26 (12.5%)	2 (5.1%)	0.27
Before the first detection of DSA^a^	26 (10.5%)	26 (12.5%)	0 (0.0%)	**0.019**
**POST-TRANSPLANTATION EVENTS**				
Delayed graft function (n, %)	72 (29.1%)	62 (29.8%)	10 (25.6%)	0.60
Rejection (n, %)	42 (17.0%)	30 (14.4%)	12 (30.8%)	**0.013**
T cell mediated rejection	40 (16.2%)	30 (14.4%)	10 (25.6%)	
Antibody mediated rejection	3 (1.2%)	0 (0.0%)	3 (7.7%)	
Time to first rejection (years)	0.5 ± 1.0	0.3 ± 0.6	1.1 ± 1.6	
Post-transplant neoplasia (n, %)	29 (11.7%)	26 (12.5%)	3 (7.7%)	0.59
Skin cancer	11 (37.9%)	11 (42.3%)	0 (0.0%)	
Hemopathy	3 (10.3%)	3 (11.5%)	0 (0.0%)	
Solid cancer	15 (51.7%)	12 (46.2%)	3 (100.0%)	
Time to post-transplant cancer (years)	3.2 ± 1.9	3.1 ± 1.9	4.0 ± 1.8	0.44
Return in dialysis (n, %)	18 (7.3%)	10 (4.8%)	8 (20.5%)	**0.003**
Death with a functioning graft (n, %)	22 (8.9%)	19 (9.1%)	3 (7.7%)	1.00
Graft failure from any cause including death (n, %)	40 (16.2%)	29 (13.9%)	11 (28.2%)	**0.027**
Mean Absolute Deviation (%)				
Continuous	19.9 ± 9.6	19.8 ± 9.3	20.7 ± 11.5	0.99
< 5%	6 (2.4%)	5 (2.4%)	1 (2.6%)	1.00
< 15%	87 (35.2%)	76 (36.5%)	11 (28.2%)	0.32
> 30%	32 (13.0%)	26 (12.5%)	6 (15.4%)	0.62
> 50%	2 (0.8%)	1 (0.5%)	1 (2.6%)	0.29

*BMI* Body Mass Index*, DSA* Donor Specific Antibody*, D−/R-* Donor negative/Recipient negative*, D−/R+* Donor negative/Recipient positive*, D+/R-* Donor positive/Recipient negative*, CNI* Calcineurin inhibitors. Results with *p* value less than 5% were emphasized using bold letters

^a^Number of patients (%) with mycophenolic acid cessation during the follow-up restricted to the period before the first DSA detection in the group “de novo DSA” and during the entire follow-up in the group “no DSA”

**Table 3:** Impact of reduced exposure to CNI on the occurrence of de novo DSA in a multivariable^a^ Cox adjusted model

Event	Reduced exposure to CNI	Nb events/Nb patients	Univariate model		Multivariate model^a^	
			HR (CI 95%)	p	HR (CI 95%)	p
First detection of one DSA with MFI > 1000	No	2/81 (2.5%)	1.00		1.00	
	Yes	35/166 (21.1%)	10.43 (2.50–43.46)	**0.001**	9.77 (2.34–40.77)	**0.002**
First detection of one DSA with MFI > 6000	No	1/82 (1.2%)	1.00		1.00	
	Yes	22/165 (13.3%)	12.31 (1.66–91.47)	**0.014**	11.54 (1.55–85.93)	**0.017**
First detection of one DSA with MFI > 10,000	No	1/84 (1.2%)	1.00		1.00	
	Yes	16/163 (9.8%)	8.86 (1.17–66.92)	**0.034**	7.40 (0.97–56.31)	0.053
First detection of DSA(s) with total MFI > 6000	No	1/82 (1.2%)	1.00		1.00	
	Yes	23/165 (13.9%)	12.81 (1.73–94.97)	**0.013**	12.02 (1.62–89.25)	**0.015**
First detection of DSA(s) with total MFI > 10,000	No	1/83 (1.2%)	1.00		1.00	
	Yes	19/164 (11.6%)	10.75 (1.44–80.43)	**0.021**	9.50 (1.26–71.43)	**0.029**
First rejection	No	1/83 (1.2%)	1.00		1.00	
	Yes	11/164 (6.7%)	5.65 (0.73–43.74)	0.097	5.65 (0.73–43.74)	0.097
Post-transplant neoplasia	No	10/88 (11.4%)	1.00		1.00	
	Yes	18/159 (11.3%)	1.16 (0.53–2.52)	0.71	1.20 (0.55–2.62)	0.64
Post-transplant neoplasia (excluding skin neoplasia)	No	8/88 (9.1%)	1.00		1.00	
	Yes	10/159 (6.3%)	0.75 (0.30–1.91)	0.55	0.75 (0.30–1.91)	0.55
Return in dialysis	No	3/87 (3.4%)	1.00		1.00	
	Yes	15/160 (9.4%)	3.22 (0.93–11.22)	0.066	3.22 (0.93–11.22)	0.066
Patient survival	No	9/87 (10.3%)	1.00		1.00	
	Yes	13/160 (8.1%)	1.01 (0.43–2.38)	0.97	1.03 (0.44–2.43)	0.94
Graft survival	No	12/87 (13.8%)	1.00		1.00	
	Yes	28/160 (17.5%)	1.57 (0.80–3.11)	0.19	1.64 (0.82–3.28)	0.16

*DSA:* Donor Specific Antibody*, MFI* Mean Fluorescence Intensity. Results with *p* value less than 5% were emphasized using bold letters

^a^Multivariable analyses were performed using iterative backward selection, by forcing “reduced exposure to CNI” in the Cox model, with the following variables as candidate covariates: number of HLA mismatches, donor type (living, deceased -standard or extended criteria-), age and gender of the recipient, mycofenolic acid cessation, delayed graft function and induction therapy

Only 3 ABMR were diagnosed during follow-up. A reduced exposure to CNI tended to be associated with an increased risk of all-type graft rejections (HR = 5.65 (0.73–43.74), *p* = 0.097).

During follow-up, 18 KTRs returned to dialysis and 22 patients died with a functioning graft. A reduced exposure to CNI tended to be associated with an increased risk of return to dialysis (HR = 3.22 (0.93–11.22), *p* = 0.066) (Table 3). There was no effect on patient survival or graft loss from any cause including death. Of note, there was no significant association between a reduced exposure to CNI and post-transplant cancer (HR = 1.20 (0.55–2.62), *p* = 0.64) (Table 3). Similar results were also found after exclusion of skin cancers.

## Discussion

### Main findings

In the present study, we demonstrate that even in a low-immunological risk population of kidney graft recipients, reduced exposure to CNI was associated with an increased risk of development of de novo DSA, known to be related to poor long-term graft outcomes. Long-term CNI exposure was assessed by taking into account different time intervals for the purpose of longitudinal pharmacological follow-up. Considering that the first detection of DSA frequently compels physicians to modify immunosuppressive treatment as well as the CNI target level, we deemed of value to take into account CNI exposure only in the period preceding DSA detection. Of note, a low exposure to CNI only tended in our cohort to be associated with increased risk of graft rejection, as well as increased risk of return to dialysis.

### CNI minimization and graft or patient prognosis

It is currently extremely difficult to draw definitive conclusions from the multiplicity of studies on CNI minimization given that strategies may vary in terms of: 1) the study population (baseline immunological risk), 2) CNI minimization strategy (withdrawn; long term maintenance with dose reduction; complete avoidance), 3) time of minimization (de novo; in case of graft function deterioration), 4) combination with an induction therapy, 5) combination with (or replacement with) maintenance therapy based on mycophenolic acid, mTOR inhibitors or belatacept. In a recent meta-analysis, Sawinski et al. assessed the impact on patient and allograft survival of four strategies of reduced-exposure to CNI (minimization, conversion, withdrawal and avoidance) [12]. The analysis of the 19 studies in which CNI minimization was associated with mycophenolic acid formulations reported reduced graft loss with this strategy, with a high level of evidence. In the most recent Cochrane meta-analysis (including randomized controlled trials (RCTs) with CNI withdrawal, tapering or low dose) [18], low dose CNI with induction regimens reduced acute rejection and graft loss, also in the short-term. The authors indicate that these conclusions must be tempered by the lack of long-term data in most of the studies, particularly with regards to chronic ABMR.

### Deleterious impact of DSA on graft prognosis

Among sensitized patients, the deleterious impact on graft survival of preformed DSA is well established [19–21], with increased risk of ABMR and graft loss. Among non-sensitized patients, dnDSA may also develop after transplantation in 15% of kidney recipients [22–24], leading to increased risk of acute rejection [23, 25] and graft loss [24, 25]. The incidence of acute rejection in kidney allograft recipients with dnDSA can reach 50%, with up to 30% subclinical acute rejection [23–25]. ABMR represents a substantial proportion of these 50% rejections [23–25], which constitutes the principal risk factor of graft loss [23–25]. dnDSA are also associated with subclinical histological lesions [26], which are an important determinant of graft survival [27, 28].

### CNI minimization and DSA development in the literature

A few previous studies have assessed the impact of CNI minimization on dnDSA development. In a recent RCT, Gatault et al. [29] evaluated the efficacy and safety of two different doses of tacrolimus in KTRs between 4 and 12 months after transplantation. Stable steroid-free patients were randomized after 4 months: Group A had a 50% reduction in tacrolimus dose with a targeted trough level > 3 ng/mL while group B had no change in tacrolimus dose (C0 7–12 ng/mL). The primary outcome was eGFR at 1 year. Estimated GFR was similar in both groups at 12 months, while dnDSA appeared only in group A (6 vs. 0 patients, *p* = 0.008). The authors concluded that tacrolimus trough levels should be maintained > 7 ng/mL during the first year after transplantation in low-immunological risk, steroid-free patients receiving mycophenolic acid.

In patients highly selected for a low immunological risk of rejection (long-term stable KTRs with no histological abnormality and absence of anti-HLA immunization), Dugast et al. conducted a double-blind RCT to analyze the benefits and risks of tacrolimus weaning [30]. Fifty-two patients were scheduled in each treatment arm, although only 10 patients were eligible and thus randomized. In the tacrolimus maintenance arm, graft function remains stable in all patients with no occurrence of graft rejection or anti-HLA immunization. In contrast, all the five patients of the placebo group developed either an acute graft rejection (humoral or not) or anti-HLA antibodies (DSA or not).

In a recent work, Béland et al. observed that among KTRs with dnDSA, higher CNI levels predicted better kidney graft survival, with a threshold of 5.3 ng/mL seemingly predictive of graft loss [16].

### Optimal trough levels of CNI / CNI target levels: Literature data

The optimal CNI trough target level remains to be defined. The KDIGO guideline suggests that maintenance immunosuppressive medication should be administered at the lowest planned dose by 2–4 months after transplantation if no rejection has occurred, although no target levels were proposed [31]. Over time, tacrolimus exposure levels have declined [31]. In more recent RCTs, the standard tacrolimus trough level 6 months after transplantation was defined between 5 and 10 ng/mL [32] or 6–9 ng/mL [33] although lower exposure ranges have been tested [34, 35]. In the present study, we consequently endeavored to encompass the latter with the term “international targets” comprised of a tacrolimus trough level between 6 and 8 ng/mL after 6 months and between 5 and 8 ng/mL after 12 months.

Certain authors have attempted to combine the data [36] of three large RCTs (the FDCC [37], Elite-Symphony [34] and OptiCept [38] trials) in order to determine the optimal tacrolimus C0 to prevent acute rejection during the first year of kidney transplantation. In general, these patients had a low-to-medium immunological risk. In the FDCC study, the mean tacrolimus C0 was 10–14 ng/mL in the first month, and tapered gradually thereafter. In the Elite-Symphony study, tacrolimus trough levels were 3-7 ng/mL during the study period. In the OptiCept trial, the tacrolimus trough levels were 8–12 ng/mL during the first month, 4–6 or 8–10 ng/mL until the end of the third months, and 3–5 or 6–8 ng/mL from the fourth month thereafter, according to the randomization groups (reduced or standard CNI dosing). Despite this pooled analysis, the authors failed to find any significant correlations between tacrolimus trough levels and the incidence of acute rejection at the different time points.

In a recent study reporting on the pooled data [39] of four RCTs [40–43] (*n* = 528) in which patients received reduced tacrolimus dosing combined with mycofenolic acid, the authors concluded that tacrolimus levels < 4 ng/mL should be avoided during the first 12 months post-transplantation when tacrolimus is used in combination with fixed-dose mycofenolic acid with or without corticosteroids and induction therapy.

### Study limitations

Certain limitations of this study should be acknowledged, the first of which being its observational single-center design. Second, protocol biopsies were not performed, nor was a biopsy systematically performed in instances of dnDSA detection. Consequently, the low number of ABMR should be taken with caution. Third, although IPV was used herein as a proxy of patient adherence [44], it remains quite difficult to distinguish true minimization from non-adherence. Nevertheless, patients with > 50% non available trough levels were excluded from the analysis, with is also a well-known marker of non-adherence among KTRs. Consequently, it would appear reasonable to assume that the proportion of non-adherent patients was low in this study. Moreover, we discuss in this study the impact of reduced exposure to CNI, irrespective of its cause. Of note only 7 patients were excluded from study population because they had ≥50% of CNI trough levels non-available. And among these patients, only one developed dnDSA during follow-up. Due to this very small number of patients, it seems unreasonable to add a third group “non-adherence” in this study. Indeed, the addition of this third group is unlikely to allow us drawing reliable conclusion. Fourth, certain other factors potentially contributing to dnDSA development were not taking into account in this study, namely post-transplant pregnancies and transfusions [45]. Finally, while multivariable analyses were adjusted according to induction treatment and mycofenolic acid cessation during follow-up, mycofenolic acid dosage (area under curve) was not taken into account.

### Clinical implications

In kidney graft recipients with low immunological risk, it is generally acknowledged that immunosuppressive treatment should be minimized given the low risk of graft rejection, and thus avoid exposing these patients to an accrued risk of neoplastic or infectious complications, as well as nephrotoxicity. As a result, clinicians frequently target low CNI trough levels in these cases. However, it should be kept in mind that such strategy is not without challenges in terms of risk of dnDSA development even in non-sensitized patients. At the very least, patients should be carefully monitored for DSA detection, in order to readjust treatment. Nevertheless, a low exposure to CNI only tended in our cohort to be associated with increased risk of graft rejection, as well as increased risk of return to dialysis. Although this absence of significant associations may be partly due to the size of our cohort, it also suggests that conflicting effects of CNI minimization might result in overall neutral effect. Despite an increased risk of dnDSA development, CNI minimization may well be beneficial for long-term graft prognosis by the way of nephrotoxicity avoidance among low-immunological risk patients [12, 18]. There are promising alternative strategies, such as the use of belatacept, which is a nonnephrotoxic drug, with no reported increased risk of DSA development [46]. The association of low-dose CNI with mTOR inhibitors could also be interesting, and has to be evaluated in regards to the risk of dnDSA development. In the recent multicenter non-inferiority trial TRANSFORM [47], 2037 de novo kidney transplant recipients were randomized to receive, everolimus with reduced-exposure CNI or mycophenolic acid with standard-exposure CNI. DnDSA incidence at 12 months and ABMR rate did not differ between the two arms. Long-term results of the studies TRANSFORM [47] and ATHENA [48] will provide useful data.

## Conclusions

Even in a low-immunological risk population, reduced exposure to CNI is associated with increased risk of dnDSA. Benefits and risks of under-immunosuppression must be carefully evaluated before deciding on CNI minimization.

## Abbreviations

ABMR: Acute antibody-mediated rejection
C0: Trough level
CNI: Calcineurin inhibitors
dnDSA: De novo Donor Specific Antibodies
ECD: Expanded criteria donors
eGFR: Estimated Glomerular Filtration Rate
HLA: Human Leukocyte Antigen
HR: Hazard Ratio
IPV: Intra-patient variability
KTR: Kidney transplant recipients
MAD%: Mean absolute deviation percent
RCT: Randomized Controlled Trial
SCD: Standard criteria donors
